# A Call for Common Sense in the Use of Molecular Imaging

**DOI:** 10.2967/jnumed.125.270550

**Published:** 2025-09

**Authors:** Ida Skarping, Thuy Tran, Rimma Axelsson, Renske Altena

**Affiliations:** 1 Lund University, Lund, Sweden; and; 2 Karolinska Institutet, Stockholm, Sweden

**TO THE EDITOR:** Molecular imaging and theranostics enhance oncologic management via integrated diagnostic and therapeutic modalities. Amid the dynamic and increasingly complex oncologic landscape, the Common Sense Oncology (CSO; commonsenseoncology.org) initiative emphasizes patient-centered care, responsible innovation, clinical benefit over hype, and rational use of new treatments and technologies. With this letter, we plead for the recognition of common sense in the field of molecular imaging.

**1. Focus on Clinical Benefit over Technologic Hype.** A core CSO principle is that innovation should yield meaningful clinical impact rather than merely demonstrate technologic sophistication. Accordingly, the use of novel PET radiotracers—for example, those targeting gastrin-releasing peptide receptor or fibroblast activation protein inhibitor—is justified when it enhances decision-making or outcomes. Linking radiotracers to therapeutic targets and an underlying disease biology strengthens their relevance and potential value. Specifically, trials focusing on the development of new tracers should focus on endpoints that generate robust evidence that can have a clinically meaningful impact.

**2. Avoid Overdiagnosis and Overtreatment.** Aligned with the principle of clinical benefit, the next CSO principle cautions against interventions that lack a clear advantage for patients, reflecting the ethical imperative to “first, do no harm.” This includes minimizing radiation exposure and avoiding the unintended consequences of highly sensitive imaging, such as detecting metastases that may shift staging without impacting treatment or prognosis, potentially leading to anxiety, overtreatment, or unnecessary labeling.

CSO advocates for an individualized imaging strategy, emphasizing careful consideration of timing (e.g., diagnostic work-up, monitoring, and scan intervals), methods (e.g., modality and tracer selection), and scope (e.g., whole-body vs. targeted imaging). This ensures a balance between prognostic accuracy and clinical relevance.

**3. Use Resources Responsibly and Equitably.** The next CSO principle emphasizes the need for fair, cost-effective resource allocation and consideration of environmental sustainability in imaging practices. Equitable access to radiopharmaceuticals and their precursors is essential for maintaining effective and inclusive molecular imaging. The global ^99m^Tc shortage in late 2024—caused by simultaneous reactor outages—underscored the vulnerability of the medical isotope supply chain and its central role in diagnostic imaging.

Furthermore, molecular imaging strategies should be evaluated for health economic impact, weighing their additional costs against potential benefits, such as treatment individualization and—when possible—de-escalation. The ZEPHIR trial, for example, demonstrated the value of targeted imaging in managing patients with breast cancer (1).

**4. Patient-Centered Decision-Making.** Patients should be actively involved in clinical decision-making, including engaging in informed discussions about incidental findings detected during imaging and contributing to clinical trial design. Initiatives such as the Choosing Wisely campaign (https://www.choosingwisely.org) provide valuable frameworks for supporting shared decision-making.

The imaging community has a responsibility to report incidental findings accurately and communicate them clearly, while remaining vigilant about the risk of treatment delays due to overinterpretation or unwarranted follow-up of clinically insignificant findings.

**5. Embrace Pragmatism and Evidence-Based Use.** At their core, actions should be driven by real-world evidence and pragmatic considerations. This highlights the importance of embedding imaging within clinical trials that prioritize robust, patient-relevant endpoints over surrogate markers.

A compelling example is the PHERGain trial in early breast cancer, where imaging is used to guide therapy de-escalation based on response, with outcomes measured through long-term, disease-specific endpoints (2).

## Final Thoughts

The implementation of new imaging tracers should be grounded in demonstrated clinical relevance, alignment with established biomarkers, and a biologic rationale and, where possible, validated through endpoints that demonstrate proven impact on patient outcomes. Standardization of image acquisition protocols and interpretation criteria—such as defining tracer avidity in relation to target expression—is critical to ensure consistency and comparability across trials.

As oncology moves toward increasingly precise and personalized care, a balanced, pragmatic approach to molecular imaging is essential. Although emerging diagnostics, such as circulating tumor DNA, show potential for real-time monitoring and may complement—or in some cases replace—imaging, clinically actionable frameworks are not yet fully developed. In this evolving landscape, it is vital to maintain a focus on clinical utility, ensuring that innovation in imaging translates into meaningful, patient-centered outcomes.

## DISCLOSURE

No potential conflict of interest relevant to this article was reported.
